# Toward fairness in digital consumer protection: a comparative legal and socio-regulatory perspective on digital platform governance

**DOI:** 10.3389/fsoc.2026.1828884

**Published:** 2026-07-22

**Authors:** Rattaakkhatee Akkhateerathitiphum

**Affiliations:** Faculty of Social Sciences, Srinakharinwirot University, Bangkok, Thailand

**Keywords:** algorithmic transparency, consumer protection, digital fairness, digital governance, platform economy, regulatory governance

## Abstract

The rapid expansion of digital platforms has transformed consumer markets and generated new regulatory challenges for consumer protection. Traditional consumer protection laws, originally designed for physical markets and bilateral contractual relationships, are increasingly inadequate to address the structural complexities of digital ecosystems characterized by algorithmic decision-making, data-driven business models, and cross-border transactions. This study examines the evolving role of fairness as a regulatory principle in digital platform governance through a comparative legal and socio-regulatory analysis of selected jurisdictions: Thailand, the United States, Canada, Spain, and Japan. In this study, fairness is understood as a normative legal principle requiring transparency of platform operations, accountability of digital intermediaries, and effective mechanisms for consumer redress in digital markets. Using doctrinal and comparative legal analysis, the research evaluates how different regulatory models address structural asymmetries between platforms and consumers. The findings reveal a global regulatory convergence toward fairness-oriented governance frameworks emphasizing transparency obligations, intermediary accountability, and accessible dispute resolution mechanisms. Based on this comparative synthesis, the study proposes the Regulated Digital Fairness Model (RDFM) as a conceptual framework integrating legal, technological, and institutional dimensions of digital governance. The study suggests that embedding fairness within digital regulation may strengthen consumer trust, enhance regulatory legitimacy, and support more sustainable digital market development, particularly in emerging digital economies such as Thailand.

## 1 Introduction

The rapid evolution of digital platforms has transformed global economic and legal structures, reshaping traditional models of commerce, consumer interaction, and market regulation. The convergence of technology and trade has generated complex regulatory challenges concerning fairness, accountability, and the protection of consumer rights in online environments (OECD, 2024; Helberger et al., 2024). Platforms such as Amazon, Shopee, Grab, and Airbnb increasingly control information flows, consumer data, and access to digital markets (Quarta, 2020). This concentration of power creates asymmetric relationships between platforms and consumers, exposing limitations in traditional consumer protection frameworks (OECD, 2024; Quarta, 2020). This study contributes to the growing literature on digital governance by examining fairness as a regulatory principle guiding digital market governance.

In this study, fairness is understood as a regulatory principle requiring transparent platform operations, accountability of digital intermediaries, and effective mechanisms for consumer redress. Within digital markets characterized by algorithmic decision-making and data-driven business models, fairness functions not only as a moral value but also as a legal standard guiding regulatory intervention to prevent exploitative or manipulative market practices.

From a theoretical perspective, digital platform regulation must be understood within the broader framework of regulatory governance and algorithmic accountability. Effective regulation requires adaptive mechanisms capable of responding to technological change (Baldwin et al., 2012), while legitimacy and accountability remain essential in polycentric governance systems (Black, 2008). In this context, fairness functions as a normative legal principle balancing market efficiency with consumer protection (Helberger et al., 2024; OECD, 2024). It encompasses algorithmic transparency, equitable contractual arrangements, and safeguards against exploitative practices in digital markets.

Thailand’s digital economy illustrates the tension between technological innovation and regulatory capacity. Although digital commerce contributes significantly to economic growth, existing legislation such as the Consumer Protection Act B. E. 2,522 was developed for traditional markets and lacks provisions addressing algorithmic decision-making, cross-border transactions, and platform liability (Nanthaphol, 2022). Consequently, Thai consumers remain vulnerable to misinformation, weak dispute resolution mechanisms, and misuse of personal data.

In contrast, jurisdictions including the United States, Canada, Spain, and Japan have developed more integrated regulatory frameworks that connect consumer protection with data governance and competition law (Bennet, 2022; Ministry of Economy, Trade and Industry (METI), 2021). These systems emphasize transparency obligations, accountability mechanisms, and specialized regulatory oversight. Examining these regulatory models provides useful comparative insights into how different legal systems institutionalize fairness within digital platform governance. Drawing on comparative legal analysis and socio-legal perspectives, this study argues that Thailand must modernize its consumer protection framework by embedding fairness as a central legal principle in digital platform governance.

To provide a meaningful comparative perspective, this study examines regulatory approaches in five jurisdictions: Thailand, the United States, Canada, Spain, and Japan. These jurisdictions were selected not only because they represent different regulatory traditions, but also because they illustrate distinct approaches to addressing platform accountability, consumer protection, and digital market governance. The United States represents an enforcement-centered model, Canada reflects an integrated approach linking consumer protection, privacy, and competition law, Spain demonstrates the operation of supranational European regulatory frameworks, while Japan provides an example of transparency-based governance. Thailand serves as the focal jurisdiction for evaluating how these regulatory approaches may inform legal reform in emerging digital economies. Comparing these models enables a more systematic assessment of how fairness is translated into regulatory institutions and enforcement practices.

### 1.1 Research gap

Although the concept of fairness has gained increasing prominence in discussions of digital governance, literature has yet to develop a comprehensive framework explaining how fairness can function as a structural principle for regulating platform economies. Much of the existing scholarship focuses on individual dimensions of digital regulation, such as data protection, competition policy, or consumer protection, without fully integrating these elements within a coherent conceptual framework for digital market governance.

In addition, comparative legal analyses examining how different jurisdictions institutionalize fairness within digital platform regulation remain relatively limited. Existing studies often emphasize specific regulatory instruments or national policy reforms rather than systematically comparing the institutional and legal mechanisms through which fairness is operationalized across legal systems.

Existing scholarship approaches fairness from different perspectives. Howells (2005) and Helberger et al. (2024) primarily examine fairness as a consumer protection principle addressing power imbalances within digital markets, while emphasizes transparency and accountability in algorithmic governance. Further situate fairness within broader debates on digital governance and regulatory legitimacy. Although these studies provide valuable theoretical insights, limited attention has been paid to how fairness is operationalized across different legal systems and institutional settings. As a result, the relationship between fairness, regulatory design, and digital consumer protection remains insufficiently explored from a comparative perspective.

This study addresses these limitations by developing a conceptual framework that situates fairness at the center of digital consumer protection and by conducting a comparative legal analysis of regulatory approaches in selected jurisdictions. This approach clarifies how fairness operates not merely as an abstract normative concept but as a practical regulatory principle embedded within legal institutions and governance mechanisms of consumer protection.

## 2 Conceptual framework

**Figure 1:**
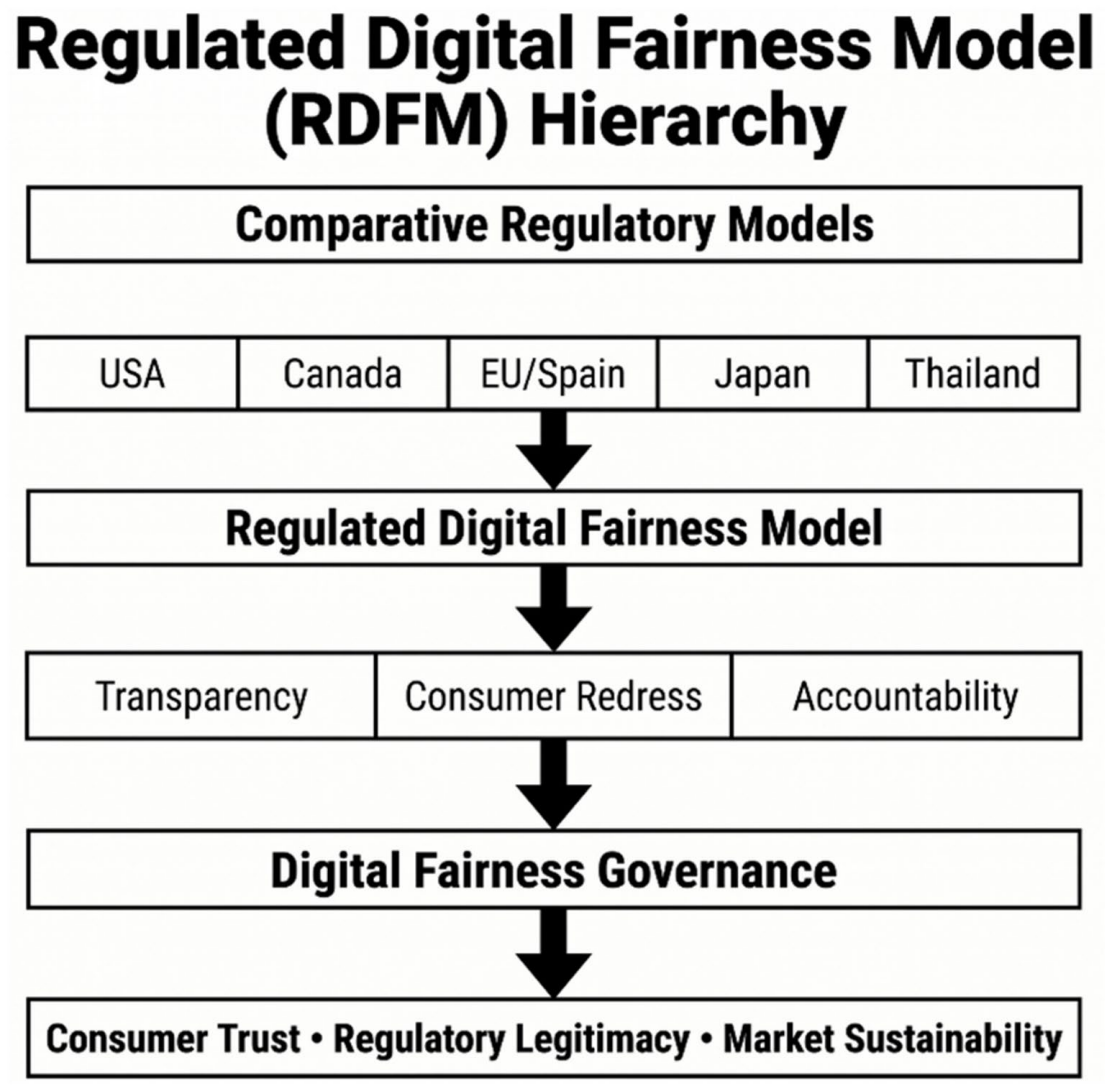
Regulated digital fairness model (RDFM): analytical dimensions of digital fairness and their relationship to digital governance outcomes.

The conceptual framework of this study integrates regulatory governance theory, law and technology scholarship, and comparative consumer protection principles. Within this framework, fairness is conceptualized as a dynamic legal norm mediating the relationship between innovation, efficiency, and accountability in digital platform regulation (Helberger et al., 2024; OECD, 2024). In this context, fairness refers to a regulatory condition in which digital platforms operate under transparent governance structures, assume responsibility for algorithmic and transactional outcomes, and provide accessible mechanisms for consumer redress (Figure 1).

### 2.1 Theoretical foundation: regulatory governance and digital fairness

Digital platforms increasingly operate as hybrid entities that function simultaneously as private intermediaries and quasi-public infrastructures regulating access to markets, information, and participation through algorithmic systems. In such environments, traditional doctrines of contract and tort often prove insufficient to address structural power asymmetries.

Regulatory governance theory provides an analytical framework for examining how states maintain legitimacy and accountability in regulating these systemically significant actors (Black, 2008). Effective regulation therefore requires reflexive legal mechanisms capable of adapting to technological change and evolving social conditions (Baldwin et al., 2012). Within this perspective, fairness functions as a regulatory meta-norm requiring transparency, predictability, and legitimacy in regulatory processes (Helberger et al., 2024). In practical terms, fairness involves obligations for platforms to disclose algorithmic processes, prevent exploitative contractual arrangements, and provide accessible dispute resolution mechanisms. Unlike traditional notions of fairness in consumer law, which primarily focus on the fairness of individual transactions, digital fairness functions as a broader regulatory principle shaping the design, implementation, and enforcement of digital governance frameworks. In this sense, fairness operates not only as a substantive consumer right but also as a mechanism for institutionalizing transparency, accountability, and consumer protection within platform ecosystems.

### 2.2 Operational dimensions of the framework

The operationalization of digital fairness is structured around three interrelated dimensions: legal adequacy and enforcement, consumer redress and accountability, and transparency and information disclosure. This framework operates fairness through three interrelated regulatory dimensions: legal adequacy and enforcement, consumer redress and accountability, and transparency in digital platform governance.

#### 2.2.1 Legal adequacy and enforcement

Legal adequacy assesses whether legal frameworks adequately address digital market realities such as algorithmic bias, cross-border services, and data-driven business models. Digital platforms create embedded power asymmetries through algorithmic design and control over information (Zuboff, 2019), requiring regulatory approaches that move beyond reactive enforcement toward adaptive and risk-based monitoring (Baldwin et al., 2012; OECD, 2024). In Thailand, enforcement remains fragmented, with agencies such as the OCPB and ETDA operating under separate mandates, thereby limiting regulatory effectiveness.

#### 2.2.2 Consumer redress and accountability

Consumer protection in digital markets requires accessible mechanisms for dispute resolution and clear allocation of responsibility. Accountability must extend beyond sellers to include digital platforms that facilitate transactions (Helberger et al., 2024). Jurisdictions with defined intermediary liability frameworks, such as Japan and the European Union, demonstrate higher levels of consumer trust (European Union, 2022). In Thailand, however, dispute resolution remains limited to administrative complaints and court procedures, while the absence of online dispute resolution (ODR) mechanisms restricts access to justice.

#### 2.2.3 Transparency and information disclosure

Transparency functions as both a preventive and corrective regulatory tool. Disclosure obligations concerning platform policies, pricing algorithms, ranking systems, and data practices allow consumers to make informed decisions and reduce manipulative behavior (Howells, 2005; Helberger et al., 2024). While jurisdictions such as Japan and Spain have institutionalized transparency through specific legislation, Thailand’s transparency requirements remain dispersed across several statutes, including the Personal Data Protection Act B. E. 2,562 and the Consumer Protection Act B. E. 2,522, resulting in limited regulatory coherence.

These three dimensions serve not only as conceptual components of digital fairness but also as analytical criteria for the comparative assessment undertaken in this study. The regulatory frameworks of the selected jurisdictions are evaluated according to their capacity to promote transparency, ensure accountability, and provide effective consumer redress. This analytical structure forms the basis of the Regulated Digital Fairness Model (RDFM). The contribution of the RDFM lies in its integration of legal, institutional, and technological dimensions of digital governance within a single analytical framework. Rather than examining transparency, accountability, or consumer redress separately, the model explains how these dimensions interact to operationalize fairness across different regulatory systems.

### 2.3 Comparative legal models as independent variables

Comparative analysis identifies regulatory models that shape digital consumer protection outcomes. Four principal models are examined.

The United States represents an enforcement-centered model relying on broad regulatory authority of institutions such as the Federal Trade Commission to address unfair digital practices through deterrence and ex post enforcement (Bennet, 2022). Canada adopts an integrated governance approach combining privacy protection and competition law, particularly through PIPEDA and the Competition Act, recognizing that informational asymmetry creates market power. Within the European Union, including Spain, fairness is institutionalized through harmonized frameworks such as the Digital Services Act and Digital Markets Act, which combine transparency, accountability, and competition regulation (European Union, 2022). Japan employs a transparency-oriented governance model under the Act on Improving Transparency and Fairness of Digital Platforms (2021), emphasizing preventive disclosure and cooperative regulatory oversight (Ministry of Economy, Trade and Industry (METI), 2021).

These models illustrate diverse regulatory cultures and institutional arrangements that collectively inform the analysis of Thailand’s digital consumer protection framework and its potential reform trajectory. These comparative models function as explanatory variables within the RDFM framework, providing the analytical basis for assessing how different regulatory systems operationalize transparency, accountability, and consumer redress in digital platform governance.

The model illustrates how comparative regulatory approaches from selected jurisdictions inform three core dimensions of digital fairness; transparency, accountability, and consumer redress; which collectively contribute to digital fairness governance and support consumer trust, regulatory legitimacy, and sustainable digital market development.

## 3 Research methodology

This study adopts a doctrinal and comparative legal research methodology to examine regulatory approaches to digital platform governance. Doctrinal analysis is employed to interpret statutory provisions, legal principles, and regulatory frameworks relevant to digital consumer protection, while comparative legal analysis is used to identify similarities and differences across selected jurisdictions (Hutchinson and Duncan, 2012; Banakar and Travers, 2023; Zweigert and Kötz, 1998).

The jurisdictions examined include Thailand, the United States, Canada, Spain, and Japan, selected because they represent diverse regulatory traditions and regulatory models of digital platform governance. Primary sources include statutory instruments, regulatory reports, and official policy documents, including the U.S. Federal Trade Commission Act, Canada’s PIPEDA and Competition Act, Spain’s Legislative Royal Decree 1/2007 together with the EU Digital Services Act, and Japan’s Transparency and Fairness of Digital Platforms Act (TFDPA 2021). Thai legislation examined includes the Consumer Protection Act B. E. 2,522, the Direct Sales and Direct Marketing Act B. E. 2,560, and the Personal Data Protection Act B. E. 2,562. Secondary sources consist of peer-reviewed academic literature and policy reports relevant to digital governance and consumer protection (Bryman, 2021).

## 4 Findings and results

The comparative analysis indicates that several jurisdictions have incorporated fairness-oriented regulatory principles into digital platform governance. For example, the European Union’s Digital Services Act introduces transparency obligations for large online platforms, requiring disclosure of content moderation practices, algorithmic decision-making processes, and data governance structures (European Union, 2022). Similarly, Japan has adopted legislation aimed at strengthening transparency and fairness in digital platform transactions. These regulatory developments illustrate a shift from traditional reactive consumer protection toward preventive governance mechanisms designed to reduce informational asymmetries in digital markets. These developments indicate an emerging regulatory trend toward incorporating fairness within digital governance frameworks.

### 4.1 Overview of comparative findings

The comparison across the United States, Canada, Spain (European Union), Japan, and Thailand reveals significant differences in regulatory philosophy and institutional enforcement capacity. Three principal governance paradigms emerge: enforcement centered regulation, exemplified by the United States; integrated governance models linking privacy, competition, and consumer protection, as seen in Canada and the European Union; and transparency-oriented governance, as illustrated by Japan. These regulatory paradigms reflect different strategies for addressing structural power asymmetries between digital platforms and consumers. In contrast, Thailand continues to rely primarily on general consumer protection legislation with limited adaptation to digital platform markets, resulting in fragmented enforcement and regulatory uncertainty. The findings therefore indicate that effective digital consumer protection requires not only legislative reform but also coherent institutional design.

### 4.2 United States

The United States adopts an enforcement-based regulatory model centered on the authority of the Federal Trade Commission (FTC). Through statutes such as the FTC Act and the Digital Platform Commission Act of 2023, regulators address deceptive or unfair practices primarily through ex post enforcement mechanisms (Bennet, 2022; OECD, 2024). This flexible framework allows regulators to interpret unfairness dynamically in response to technological developments. However, the reliance on ex post enforcement means that regulatory intervention often occurs only after consumer harm has materialized. Consequently, reliance on institutional discretion may also generate risks of regulatory inconsistency or regulatory capture (Baldwin et al., 2012). From the perspective of the Regulated Digital Fairness Model (RDFM), the United States demonstrates a strong accountability dimension through the broad enforcement powers of the FTC and its capacity to respond flexibly to emerging digital harms. However, compared with the European Union and Japan, the U.S. framework places less emphasis on ex ante transparency obligations. As a result, fairness is primarily achieved through enforcement and deterrence rather than through preventive regulatory design.

### 4.3 Canada

Canada adopts an integrated regulatory approach that links consumer protection, privacy law, and competition regulation. The Personal Information Protection and Electronic Documents Act (PIPEDA) and the Competition Act collectively address both informational asymmetry and market dominance (Department of Justice Canada, 1985). By integrating these domains, Canada conceptualizes digital fairness as a combination of data protection, market equality, and consumer autonomy. This integrated regulatory architecture enables regulators to address digital market failures through multiple legal instruments rather than relying on a single regulatory domain. Proactive oversight by the Competition Bureau further strengthens compliance and consumer confidence. Compared with the U.S. model, Canada adopts a more integrated regulatory approach by linking consumer protection, privacy, and competition law within a coherent governance framework. This integration strengthens both transparency and accountability, reducing the regulatory fragmentation often associated with digital platform oversight. From an RDFM perspective, Canada illustrates how fairness can be advanced through institutional coordination rather than enforcement alone.

### 4.4 Spain and the European union

Spain’s consumer protection regime reflects the broader European Union framework under instruments such as the Digital Services Act and related directives. These regulations establish transparency obligations, algorithmic accountability mechanisms, and user redress systems (European Union, 2022). Unlike enforcement-centered models, the EU approach institutionalizes fairness through detailed regulatory obligations that apply ex ante to platform governance. This multi-level governance structure harmonizes consumer protection standards across member states while facilitating cross-border enforcement. However, coordination challenges may arise between EU institutions and national authorities (Helberger et al., 2024). From an RDFM perspective, the European Union presents the most comprehensive approach among the jurisdictions examined, integrating transparency, accountability, and consumer redress within a unified regulatory framework. Unlike the U.S. emphasis on enforcement or the Japanese focus on disclosure, the EU approach combines preventive obligations with institutional enforcement mechanisms, thereby strengthening regulatory coherence in digital consumer protection.

### 4.5 Japan

Japan’s Act on Improving Transparency and Fairness of Digital Platforms (TFDPA 2021) represents a transparency-centered regulatory model emphasizing preventive disclosure and cooperative oversight (Ministry of Economy, Trade and Industry (METI), 2021). Under this framework, platforms must disclose ranking algorithms, contractual relationships, and business practices. Rather than relying primarily on punitive enforcement, the Japanese model seeks to prevent unfair practices by increasing transparency in platform governance. The system is jointly administered by the Consumer Affairs Agency and the Japan Fair Trade Commission, combining regulatory supervision with cooperative governance. This approach reduces information asymmetry and strengthens long-term consumer trust (Ministry of Economy, Trade and Industry (METI), 2021; Helberger et al., 2024). From a comparative perspective, Japan demonstrates how fairness can be promoted through transparency and cooperative governance rather than extensive regulatory intervention. Although its enforcement mechanisms are less interventionist than those found in the United States and the European Union, the emphasis on disclosure and stakeholder engagement contributes to reducing information asymmetries and strengthening consumer trust.

### 4.6 Thailand

Thailand’s digital consumer protection framework remains primarily based on traditional legislation, including the Consumer Protection Act B. E. 2,522 and the Direct Sales and Direct Marketing Act B. E. 2,560. These laws were designed for conventional commerce and do not adequately address platform-specific issues such as algorithmic liability, cross-border digital services, and data governance (Nanthaphol, 2022). As a result, regulatory authorities often encounter difficulties when addressing digital market practices such as misleading online advertising, manipulative platform interfaces, and cross-border consumer fraud.

Institutionally, the Office of the Consumer Protection Board (OCPB) faces limitations in digital investigative capacity and jurisdiction over transnational platforms. Enforcement largely relies on reactive complaint mechanisms rather than preventive regulatory strategies. This reactive enforcement structure reduces the effectiveness of consumer protection in rapidly evolving digital markets. In addition, the absence of dedicated frameworks for online dispute resolution and algorithmic transparency highlights structural gaps in consumer protection.

Consequently, Thailand faces both a normative deficit, reflected in the absence of explicit platform liability standards, and an institutional deficit caused by fragmented regulatory authority among agencies such as the OCPB, ETDA, and competition regulators. Recent developments further illustrate these challenges. The growth of cross-border e-commerce, social commerce, and online fraud has increased concerns regarding platform accountability and the effectiveness of consumer redress mechanisms. Consumers frequently encounter difficulties in identifying responsible parties, particularly where transactions involve foreign platform operators or complex digital supply chains. These developments highlight the limitations of existing legal frameworks and reinforce the need for more coherent digital consumer protection measures. From an RDFM perspective, Thailand continues to demonstrate weaknesses across the dimensions of transparency, accountability, and consumer redress, highlighting the need for both legal and institutional reform.

### 4.7 Synthesis of results: the global convergence of fairness

Comparative analysis indicates that, despite differences in legal traditions and institutional structures, fairness has emerged as a central normative principle in digital platform regulation. Increasingly, it functions as a meta-regulatory value guiding transparency, accountability, and consumer redress across jurisdictions (Helberger et al., 2024; OECD, 2024). This development reflects a broader shift in regulatory philosophy from reactive consumer protection toward proactive governance of digital market infrastructures. In this context, fairness extends beyond traditional consumer law doctrines and becomes embedded within broader digital governance frameworks.

This convergence reflects the emergence of a Digital Fairness Paradigm (DFP), which integrates economic efficiency with ethical governance. Within this paradigm, fairness evolves from a normative ideal into a practical regulatory standard implemented through institutional mechanisms such as algorithmic oversight, consumer choice architecture, and participatory regulatory processes. Consequently, fairness functions as a governance principle shaping the institutional design of digital regulation.

Two structural forces contribute to this development. First, data globalization increasingly blurs national regulatory boundaries, encouraging states to adopt interoperable fairness standards for cross-border digital markets (OECD, 2024). Second, technological standardization has enabled digital platforms to function as quasi-regulatory infrastructures requiring coordinated public oversight (Quarta, 2020; OECD, 2024). These developments further explain why fairness has become a shared regulatory language across different legal systems. Viewed through the RDFM framework, the selected jurisdictions demonstrate different pathways toward digital fairness. The United States prioritizes accountability through enforcement, Canada emphasizes institutional integration, the European Union combines transparency, accountability, and redress within a comprehensive regulatory structure, while Japan focuses on procedural transparency and cooperative governance. Thailand, by contrast, continues to exhibit weaknesses across all three dimensions, particularly in relation to institutional coordination and platform-specific regulatory safeguards.

Across jurisdictions, three recurring characteristics of digital fairness regulation can be observed: transparency through disclosure of platform policies and algorithms; accountability through expanded liability frameworks for digital intermediaries; and procedural redress through accessible complaint mechanisms and online dispute resolution systems. Together, these elements constitute the operational dimensions of fairness within digital governance. The conclusion regarding regulatory convergence is derived from the recurring presence of these three dimensions across the jurisdictions examined. Although the selected legal systems differ in regulatory traditions and institutional arrangements, each increasingly incorporates transparency obligations, accountability mechanisms, and consumer redress structures as central components of digital platform governance.

Collectively, these developments suggest that fairness is becoming a foundational governance principle in the digital economy. Comparative findings demonstrate that jurisdictions increasingly rely on fairness as a mechanism for balancing technological innovation with consumer protection and regulatory accountability. For Thailand, embedding fairness within regulatory frameworks is essential to strengthen consumer protection, enhance regulatory legitimacy, and align domestic law with evolving international standards in digital platform governance. This conclusion is supported by the increasing incorporation of transparency obligations under the EU Digital Services Act, the Japanese TFDPA, and emerging discussions on digital fairness within OECD consumer policy initiatives.

## 5 Discussion

The findings indicate that fairness has evolved from a moral aspiration into a regulatory imperative, reflecting the transformation of digital consumer protection into a multidimensional governance framework. Within regulatory theory, fairness performs a dual function: it legitimizes state intervention while simultaneously constraining excessive market power (Black, 2008; Baldwin et al., 2012). In digital markets characterized by algorithmic decision-making and rapid technological change, fairness acts as a stabilizing principle that balances innovation with consumer protection. This role becomes particularly significant in platform-based markets where digital intermediaries exercise substantial influence over information flows, pricing structures, and market access. Consistent with responsive regulation theory, regulatory legitimacy increasingly depends on adaptive and proportional regulatory mechanisms rather than rigid command-and-control approaches (Ayres and Braithwaite, 1992).

Socio-legal scholarship further emphasizes that fairness must extend beyond formal legal rules to reflect societal expectations of justice in digitally mediated environments (Cotterrell, 2018). As digital platforms increasingly function as social infrastructures shaping communication, work, and consumption (Zuboff, 2019), fairness emerges as a foundational governance value comparable to procedural safeguards in administrative law. In this respect, fairness does not merely regulate individual transactions but also addresses the structural conditions under which participation in digital markets takes place.

The comparative analysis reveals three broad patterns of regulatory evolution. First, regulatory systems are moving from fragmented legal frameworks toward integrated governance structures in which institutions such as the U.S. Federal Trade Commission and Canada’s Competition Bureau exercise broader cross-sector oversight of data governance, competition policy, and consumer protection (OECD, 2024). This shift suggests that legal coherence in digital markets increasingly depends on institutional coordination rather than on isolated statutory reform alone. Second, many jurisdictions are shifting from ex post enforcement toward preventive regulation through transparency obligations and algorithmic accountability mechanisms (Ministry of Economy, Trade and Industry (METI), 2021; European Union, 2022). The significance of this development lies in the fact that consumer protection is no longer triggered only after harm occurs but is increasingly embedded in the design of regulatory systems themselves. Third, regulatory authority increasingly operates within polycentric governance arrangements involving states, private actors, and civil society, thereby enhancing regulatory legitimacy through participatory oversight (Black, 2008; OECD, 2024).

In contrast, Thailand’s consumer protection framework remains fragmented and largely oriented toward traditional commercial transactions. Existing legislation such as the Consumer Protection Act B. E. 2,522 and the Direct Sales and Direct Marketing Act B. E. 2,560 does not adequately address algorithmic decision-making, cross-border digital services, or platform accountability (Nanthaphol, 2022). Institutional fragmentation among agencies including the OCPB, ETDA, and PDPC further limits regulatory coherence and enforcement capacity. Therefore, Thailand’s current framework struggles to respond effectively to platform-based risks that arise from data-driven transactions, opaque ranking systems, and cross-border digital intermediation.

To move toward fairness-based governance, Thailand should pursue reforms on three levels. First, legal reform should codify fairness as a guiding principle within a dedicated digital platform regulatory framework (Helberger et al., 2024). Second, institutional reform should establish an integrated regulatory authority capable of coordinating consumer protection, competition, and data governance. Third, procedural reform should expand accessible Online Dispute Resolution mechanisms and participatory consumer feedback systems, consistent with OECD recommendations for digital consumer protection (OECD, 2024). Taken together, these reforms would address not only gaps in substantive law but also weaknesses in the institutional architecture of digital consumer protection.

These reforms would strengthen regulatory legitimacy while aligning Thailand’s legal framework with evolving global standards. More broadly, the study contributes to digital governance scholarship by reconceptualizing fairness as a dynamic regulatory meta-norm and by introducing the Regulated Digital Fairness Model (RDFM) as a framework for comparative legal reform in digital platform governance. In this respect, the study not only proposes legal reform for Thailand but also provides a comparative analytical perspective on how fairness may be institutionalized across different digital regulatory systems.

## 6 Policy implications

The findings of this study suggest that Thailand’s digital consumer protection framework should move from a reactive enforcement regime toward a principle-based governance system. Drawing on the comparative analysis and theoretical insights developed in this research, several policy reforms can be identified to strengthen the governance of digital platform markets.

### 6.1 Enactment of a digital platform consumer protection act (DPCPA)

Thailand currently lacks a comprehensive legal framework specifically designed to regulate digital platform markets. A Digital Platform Consumer Protection Act could address structural gaps in existing legislation by explicitly codifying fairness as a regulatory obligation and by establishing clearer duties for platform operators regarding transparency, algorithmic accountability, and equitable contractual practices. Drawing on comparative regulatory models such as Japan’s Transparency and Fairness of Digital Platforms Act (TFDPA 2021) and the European Union’s Digital Services Act (2022), such legislation may include obligations concerning the disclosure of ranking algorithms and data governance practices, liability standards addressing digital service failures and online fraud, and administrative sanctions for regulatory non-compliance (European Union, 2022; Ministry of Economy, Trade and Industry (METI), 2021; Helberger et al., 2024).

### 6.2 Establishment of a digital market and consumer protection commission (DM-CPC)

Institutional reform is also necessary to address regulatory fragmentation among agencies such as the Office of the Consumer Protection Board (OCPB), the Electronic Transactions Development Agency (ETDA), and the Personal Data Protection Committee (PDPC), a structural issue that has also been observed in broader analyses of Thailand’s digital regulatory landscape (Chureemas, 2023). The establishment of a centralized regulatory authority could integrate consumer protection, data governance, and competition oversight within a more coherent administrative structure. Such an institution should possess investigative authority to conduct algorithmic audits, enforcement powers to impose sanctions where necessary, and advisory functions enabling structured consultation with industry stakeholders and civil society (Black, 2008; OECD, 2024). Institutional integration of this nature would likely enhance regulatory coherence while strengthening the legitimacy of digital market governance.

### 6.3 Integration of algorithmic transparency and data ethics principles

Thailand should incorporate algorithmic transparency more explicitly into its legal framework by requiring digital platforms to disclose key criteria governing product recommendations, advertising placement, and algorithmic pricing systems. In addition, the processing of consumer data should be guided by data ethics principles, including fairness, purpose limitation, and non-discrimination. These measures would strengthen the link between consumer protection and data governance, reflecting regulatory developments in Canada and the European Union.

### 6.4 Development of online dispute resolution (ODR) and digital mediation platforms

Accessible dispute resolution mechanisms represent an essential dimension of procedural fairness in digital markets. Thailand could therefore develop national Online Dispute Resolution (ODR) systems integrated with e-commerce platforms to enable efficient and low-cost resolution of consumer disputes. Such systems may incorporate digital mediation mechanisms, multilingual accessibility for regional users, and institutional arrangements linking ODR outcomes with existing judicial enforcement procedures. This approach aligns with international standards such as the UNCITRAL Technical Notes on ODR and the OECD Consumer Policy Toolkit (2024).

Taken together, these reforms support the broader proposition that fairness has become an increasingly important source of regulatory legitimacy in the digital age. Embedding fairness within legal frameworks and institutional structures would enable Thailand to move beyond compliance-based regulation toward a more adaptive, coordinated, accountable system of digital market governance (Ayres and Braithwaite, 1992; Cotterrell, 2018).

## 7 Conclusion

This study contributes to the emerging field of digital governance jurisprudence by positioning fairness as a foundational principle for regulating platform economies. In an era where algorithmic systems increasingly mediate access to markets, information, and opportunities, fairness functions as a foundational regulatory principle in the digital economy. The study’s comparative findings demonstrate that jurisdictions achieving sustainable digital growth are those that embed fairness not only within legal frameworks but also within institutional practice.

For Thailand, these findings suggest the need for a significant paradigm shift, from fragmented legal frameworks toward a more unified, fairness-oriented system of digital governance, a challenge frequently discussed in studies of Thailand’s evolving digital regulatory framework (Chureemas, 2023). Enacting a Digital Platform Consumer Protection Act, establishing an integrated regulatory authority, and institutionalizing transparency, accountability, and effective redress mechanisms represent not merely administrative reforms but steps toward the constitutionalization of fairness within Thailand’s evolving digital regulatory order.

By embracing fairness as both a normative ideal and a practical governance strategy, Thailand can strengthen consumer confidence, enhance international regulatory credibility, and contribute meaningfully to the development of a more just, transparent, and human-centered digital future.

## Data Availability

The original contributions presented in the study are included in the article/supplementary material, further inquiries can be directed to the corresponding author.
